# How materials can beat a virus

**DOI:** 10.1007/s10853-020-04678-4

**Published:** 2020-05-12

**Authors:** Samuel T. Jones

**Affiliations:** 1 Department of Materials, University of Manchester, Oxford Road, Manchester, M13 9PL UK; 2 The Henry Royce Institute, Alan Turing Building, Oxford Road, Manchester, M13 9PL UK

At the end of 2019 reports started to surface of a novel viral infection that has become known as SARS-CoV-2 (causing the infection COVID-19) and was classified as a pandemic by the World Health Organization (WHO) in March 2020. Countries around the world have utilised a range of measures to face this pandemic and although each country has their own unique approach, it has typically included periods of self-isolation or quarantine to help limit both the spread of the virus and the burden on health care systems. During these periods of self-isolation, many have been asking themselves what they can do to help. There have been some uplifting reports of everyone pulling together to do their part, whether that is breweries, distilleries or perfume manufacturers making hand sanitiser, [4] or collaborative teams of engineers and scientists working together to produce and redesign ventilators [2].

But what could a materials chemist do to help? Viruses are often thought of as being a problem to be resolved by biologists and biochemists, yet in reality, there are many crossovers with chemistry and materials, ready to be exploited [11].

Viruses are intracellular parasites with a very simple structure. They lack their own metabolism and require a host to replicate, so are (debatably) not living [8]. Instead viruses can be thought of as self-assembled nanostructures, typically comprised of proteins, genetic material, and often a lipid membrane. Prof. Palli Thordarson (Department of Chemistry, The University of New South Wales (UNSW)) recently highlighted this in a Twitter thread [13], showing why it is so important to use soap when washing your hands to destroy viruses like SARS-CoV-2.

**Figure 1:**
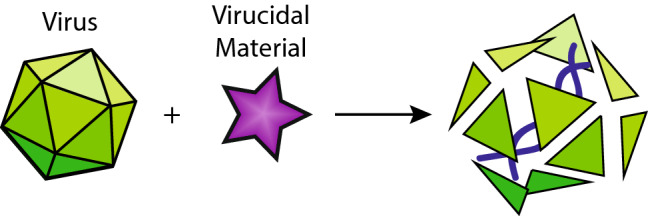
Cartoon depicting the affect a virucidal material has on a virus

The key characteristic behind why soap is so effective is linked directly to the fact that viruses are self-assembled structures. Their lipid membranes are comprised of fatty acids with hydrophobic interactions between chains, leading to a stable outer membrane. When you introduce soap into the mixture these hydrophobic interactions are disrupted and the lipid membrane is ‘dissolved’, destroying or deactivating the virus. Materials capable of destroying a virus in such a way are termed virucidal (Fig. 1) and there are many examples: bleach, alcohol and, as already mentioned, soap. This is a simple approach to destroying a virus and it is widely used in many areas from virology labs, (Virkon—used to sterilise and decontaminate surface) through to hospitals (alcohol hand gel). Yet, it is not possible to use soap (or other virucides) as an internal treatment because the exact same process that destroys the virus would also destroy our own cells.

On the topic of treatment options for viruses, there are two main approaches to antivirals, intracellular and extracellular, both of which can benefit greatly from input from materials chemists.

**Figure 2:**
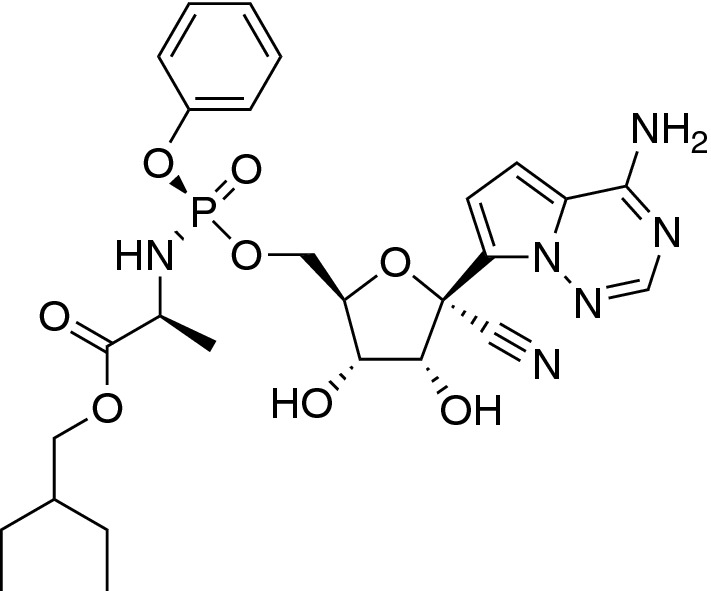
Chemical structure of Remdesivir, a potential antiviral candidates for treatment of COVID-19

Intracellular antivirals are almost exclusively small-molecule drugs that are designed to inhibit the intracellular replication of viruses, for example some of the promising candidates for treating SARS-CoV-2: Remdesivir (Fig. 2), which was originally developed for Ebola [14], and Kaletra (ritonavir and lopinavir combination), originally designed for HIV [1].

As with the majority of these intracellular drugs, they are designed to be effective against a specific virus and although they may be effective against other viruses, there are no guarantees. To ensure success, each drug would need to redesigned using, for example, in silico studies or a process of trial and error; however, this would mean a re-design for every viral outbreak. An intracellular approach does not readily lend itself towards a broad-spectrum drug, because each virus utilises a different replication pathway to multiply.

A broad-spectrum antiviral, similar to broad-spectrum antibiotics, would rapidly treat newly emerging viral outbreaks. Extracellular antivirals, unlike intracellular antivirals, become important here as they have potential to be the most broad spectrum. There are many types of extracellular antivirals, but the most common are ‘entry inhibitors’—another area where materials chemists can get their hands dirty.

Entry inhibitors are, in general, materials that bind to the virus surface and block the interactions with the host cells, thereby stopping the virus from being able to infect. As different viruses can use the same (or similar) initial attachment receptors on the surface of cells [5]; these entry inhibitors can be designed to mimic such receptors. In doing so, these materials show broad-spectrum activity with other viruses that use the same initial attachment receptors.

In principal, these sound like our best approach; however, the interaction between material and virus can be reversible (termed virustatic) and so with a simple change in dilution, pH or temperature the infective virion would be released. There have been many examples of broad-spectrum virustatic materials including polymers [9, 12], dendrimers [10], and nanoparticles [3] and all show very low levels of toxicity. Yet, when it comes to their use as a treatment, these virustatic materials fall short because in the *in vivo* environment dilution occurs readily, leading to release of the intact virion [12].

What if we could take these non-toxic, broad-spectrum entry inhibitors, modify them and change their mode of action from virustatic to virucidal? This change would lead to a material that was able to destroy a wide range of viruses on contact (i.e. broad spectrum) while remaining non-toxic. This might sound too good to be true, but my colleagues and I recently discovered materials capable of doing just that [6, 7].

**Figure 3:**
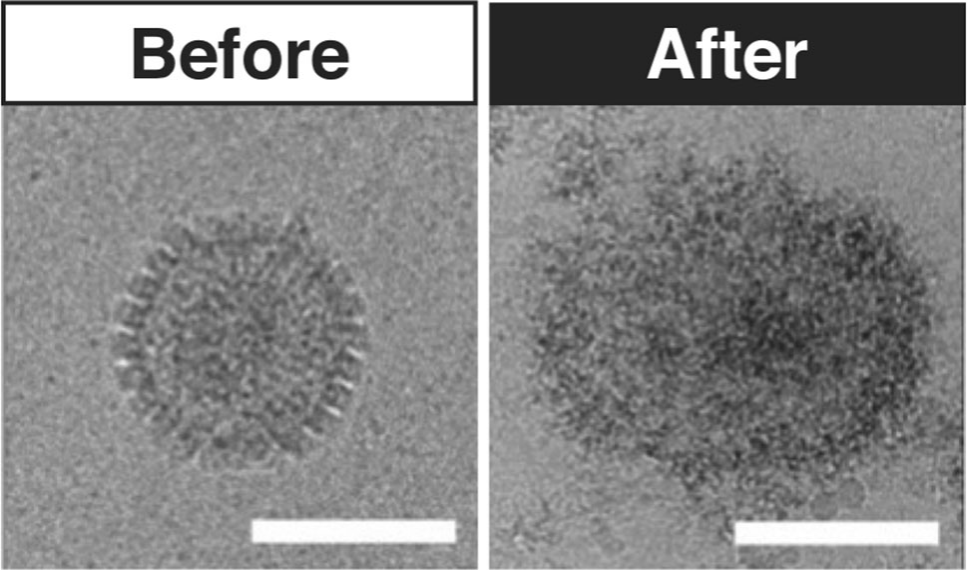
Cryo-transmission electron microscopy images of the Herpes Simplex Virus 2 (HSV-2) before (left) and after (right) interacting with the virucidal cyclodextrin variant. Scale bar = 100 nm

By modifying a sugar macrocycle, called cyclodextrin, with alkyl sulfonates, we were able to show broad-spectrum virucidal effects against viruses that use cell-surface heperan sulfates as their initial attachment receptors (Fig. 3). These include, but are not limited to, human immunodeficiency virus (HIV) ($\hbox {EC}_{{50}} = 3.35\,\upmu \hbox {M}$), herpes simplex virus 1 (HSV-1) ($\hbox {EC}_{{50}} = 15.3\,\upmu \hbox {M}$), herpes simplex virus 2 (HSV-2) ($\hbox {EC}_{{50}} = 8.61\,\upmu \hbox {M}$), respiratory syncytial virus A (RSV-a) ($\hbox {EC}_{{50}} = 2.99\,\upmu \hbox {M}$), Dengue virus ($\hbox {EC}_{{50}} = 4.63\,\upmu \hbox {M}$), Zika virus ($\hbox {EC}_{{50}} = 0.2\,\upmu \hbox {M}$) and hepatitis C ($\hbox {EC}_{{50}} = 2.04\,\upmu \hbox {M}$). As well as being broad spectrum, these modified cyclodextrins were virucidal and showed very low toxicity in vitro. We observed that the linker length between the cyclodextrin backbone and the sulfonate group was critical for achieving this virucidal mechanism, with shorter alkyl linkers leading to poorer EC50’s and loss of the virucidal mechanism.

Through the use of molecular dynamic simulations, we show that upon binding of the modified cyclodextrins to the attachment receptor of the HSV2 virus, the protein trimer is altered only in the presence of the virucidal cyclodextrin variant.

Initial tests of these antivirals against SARS-CoV-2 has shown promising efficacy, but further tests are needed to confirm this finding. The key now is to try and develop these types of materials, not only for clinical use in the fight against SARS-CoV-2 (if needed), but also ultimately so that they are ready for the next inevitable viral outbreak.

This represents only one example of a material-based approach that can be used in the fight against viral infections. There are many other avenues waiting to be explored (such as antiviral face masks or antiviral surfaces) that offer materials chemists further opportiunities to apply their expertise to the virus problem.
